# The effects of others’ drinking on the harms to children in Thailand: Lessons from the WHO-ThaiHealth project

**DOI:** 10.1371/journal.pone.0265641

**Published:** 2022-03-23

**Authors:** Perawas Preampruchcha, Nattapong Suwanno, Butpetch Petchana, Tirada Kuemee, Athip Tanaree, Jiraluck Nontarak, Karnsunaphat Balthip, Khemanat Ratworawong, Narisa Hayiyani, Nurtasneam Oumudee, Ongart Maneemai, Orratai Waleewong, Rassamee Chotipanvithayakul, Sopit Nasueb, Supeecha Rungruang, Surasak Chaiyasong, Surasak Saokaew, Tanomsri Intanont, Teerohah Donraman, Udomsak Saengow, Warangkhana Duangpaen, Warintorn Bunyanukul, Polathep Vichitkunakorn

**Affiliations:** 1 Faculty of Medicine, Prince of Songkla University, Hat Yai, Songkhla, Thailand; 2 Department of Mental Health, Ministry of Public Health, Nonthaburi, Thailand; 3 Faculty of Public Health, Department of Epidemiology, Mahidol University, Rajthevee, Bangkok, Thailand; 4 Faculty of Nursing, Prince of Songkla University, Hat Yai, Songkhla, Thailand; 5 School of Pharmaceutical Sciences, University of Phayao, Muang Phayao, Thailand; 6 Faculty of Medicine, Department of Epidemiology, Prince of Songkla University, Hat Yai, Songkhla, Thailand; 7 International Health Policy Program, Ministry of Public Health, Nonthaburi, Thailand; 8 Faculty of Medicine, Department of Family and Preventive Medicine, Prince of Songkla University, Hat Yai, Songkhla, Thailand; 9 Social Pharmacy Research Unit, Faculty of Pharmacy, Mahasarakham University, Maha Sarakham, Thailand; 10 Faculty of Medicine, Division of Digital Innovation and Data Analytics, Prince of Songkhla University, Hat Yai, Songkhla, Thailand; 11 Center of Excellence in Data Science for Health Study, Walailak University, Tha Sala, Nakhon Si Thammarat, Thailand; 12 School of Medicine, Walailak University, Tha Sala, Nakhon Si Thammarat, Thailand; 13 Faculty of Science, Prince of Songkla University, Hat Yai, Songkhla, Thailand; Chiang Mai University, THAILAND

## Abstract

**Background:**

Many knowledge gaps exist in the area of alcohol-related harms in children research such as the potential impact of other’s drinking and their social demography. Thus, this study aims to evaluate the effects of characteristics of household members and others’ alcohol drinking on harms to children in Thailand.

**Data and methods:**

This study examined 952 parents caring for children and adolescents under 18 years of age, using the questionnaire (i.e., The Harm to Others from Drinking under the WHO/ThaiHealth International Collaboration Research Project). They were interviewed between September 2012 and March 2013.

**Results:**

The study found that 15.89% of children and young people were affected by someone’s drinking in at least one category of harms. People over 60 years of age were less likely to cause alcohol-related harm to children than those aged 18 to 29 (adjusted odds ratio [AOR] 0.19, 95% confidence interval [Cl]: 0.06–0.58). Households with a binge drinker or regular drinker (≥1 time/week) were more likely to have children at higher risk of suffering alcohol-related harm in comparison to households without alcohol drinker (AOR 4.75 and 1.92, respectively).

**Conclusion:**

This study found that children whose family members are young adult or consume alcohol (i.e., weekly drinker or binge drinker) were significantly adversely affected. The most common problems were domestic violence and verbal abuse. Most of the problems, affecting children, were caused mostly by their parents.

## Introduction

Alcohol consumption negatively affects drinkers and those around them and causes wellness losses accounting for 5.1% of the global burden of disease and injury [1], alcohol drinking also has a negative impact on society, such as crime [2], assault [3], and overall economic loss [4].

Studying the effects of alcohol consumption is important in order to better understand the conditions and patterns of the problems associated with it as well as any preventive approaches that could be implemented. At present, there are many studies that have collected the effects of alcohol consumption on drinkers, especially in regards to the health of drinkers [5]. However, alcohol consumption can also affect other individuals around the drinker. In the last 10 years, there has been more global attention paid to such research issues [6,7]. In 2010, Robin Room [8] described the effects of alcohol consumption via the impact it has on people around the drinker; such as, family members, friends, colleagues, or strangers. These effects can take the form of assault, sarcastic chatter from drinkers and/or bearing the burden of drinkers, such as caring for drinkers, working on the behalf of colleagues, or even being forced or sexually oppressed by drinkers [7].

In addition, studies on the effects of alcohol consumption on individuals around drinkers are part of the conceptual framework of the Alcohol Problem Management Mechanism in the Global Alcohol Consumption Situation Report in 2014 [9]. This demonstrated the importance of addressing the problems of global alcohol consumption [9].

Parental binge drinking affects children with a wide range of age and can result to verbal [10,11] or physical violence [10,12]; financial problems [10]; neglect or lack of quality parenting [10,13]; road accidents [12]; assistance from various agencies being required [10]; children’s own binge-drinking habits, such as having a reduced drinking age etc. [14]; murder [15]; unintentional injuries [12,16]; and depression, anxiety, Learning & Communication Problems [17].

In high-income countries (HIC), 10 to 20 percent of children were in families with alcohol use disorders (AUD) [11,17,18]. Alcohol consumption in low- and middle-income countries (LAMIC) tended to be harm and had higher proportion of heavy drinker than the HIC [19]. Alcohol-misuse in the LAMIC affected to many negative consequences such as substance use, mental health problems, teenage pregnancy, and others [20]. Moreover, about five percent of caregivers reported that their children’s needs were not satisfy due to financial harm caused by others’ drinking, particularly in the LAMIC [21]. The Harm to Others from Drinking by the WHO-ThaiHealth project [22] found that, among eight countries, Thailand had the second-largest problem of alcohol consumption in children under the age of 18, which represent 12.8% of all children. The factors associated with child exposure were family heavy drinkers, which were correlated in eight countries in the WHO-ThaiHealth project [22] and the United States [10]. The greatest impact on children was parents (49.1%), followed by relatives (22.0%) [10].

There is limited research on the effects of drinking alcohol in Thailand. A study by Waleewong et al [23] has identified the size of the problem in the national landscape. Nevertheless, in regards to descriptive and in-depth individual issues studies, there are many knowledge gaps such as the demographic characteristics of the household where the impact on children living in the household is reported. Thus, this study aims to evaluate the effects of characteristics of household members and others’ alcohol drinking on harms to children in Thailand.

## Methods

### Study setting and participants

This study was a secondary data analysis research, which approved by the Human Research Ethics Committee of the Faculty of Medicine, Prince of Songkla University, Thailand (see **Ethical consideration section** for more details). It was part of a larger project titled “The Harm to Others from Drinking” by the “WHO-ThaiHealth project”; organised by the World Health Organisation (WHO) and the Thai Health Promotion Foundation (ThaiHealth) [24]. In Thailand, the cross-sectional study was conducted from four provinces (Chiang Mai, Khonkaen, Chonburi, and Surathani) in four regions, and Bangkok. Our participants were Thai residents aged over 18 years old and living with children in their household. The participants were selected using a multi-stage cluster sampling technique. More details about the data collection can be found elsewhere [24].

### Data collection

The secondary data were collected using face-to-face interviews between September 2012 and March 2013. Each participant was interviewed using a structured questionnaire (i.e., “The Harm to Others from Drinking under the WHO/ThaiHealth International Collaboration Research Project”) by a trained interviewer. There were 11 sections in the questionnaire. It took about 20–50 minutes to answer the questions.

#### Dependent variable–Alcohol’s harm to children

Alcohol’s harm to children was identified from the experiences of participants whose children had been adversely affected by others’ drinking. A series of questions on alcohol’s harm to children were as follows:

- *“Did children witness serious violence in the home*?*”*
- *“Were children yelled at*, *criticised*, *verbally abused*?*”*
- *“Were there not enough money for the things needed by children*?*”*
- *“Were children abandoned in an unsupervised or unsafe situation*?*”*
- *“Were physically hurt*?*”*
- *“Was child protection agency or family services called*?*”*

For each question, the participants can choose one answer (Answer: 3 times or more/1-2 times/No). Then, they were asked to answer, “What was the relationship to children of that person/those people?” (Answer: parent, step-parent, sibling, father’s side cousin, mother’s side cousin, friends, others such as teacher).

#### Independent variable–presence of binge or frequent drinker in household

The main independent variable was the “**Presence of a binge or frequent drinker in household”.** In the survey, each participant was asked to identify the binge or frequent drinker in their household.

- **Binge drinking:** Participants were asked *“Thinking about the last 12 months*, *can you think of anyone else among the people in your life or others that you would consider a problem drinker or someone that drinks large amounts of alcohol often*?*”* (Answer: No, Yes No). If the participant answered “Yes”, they were asked about the relationship types (Answer: spouse/partner who stay together without marriage registration, others in household or family, other relatives such as sister-, son- and daughter-in-law, ex-spouse/ex-partner, boyfriend/girlfriend, friends, colleague, and others. Then, we defined binge drinker in household for first two relationships.
- **Frequent drinking:** Participants were asked *“How many times did you drink alcohol in the last 12 months*?*”* (Answer: Every day, five or six times per week, three or four times a week, once or twice days a week, two to three times a month, once a month, and less than once a month; never in the last 12 months, and never in the lifetime). Then we classified it into three levels (i.e., Not in the previous year, monthly or less, and weekly) and excluded the 12-month and lifetime abstainers.

### Statistical analysis

Data were analysed using program R version 4.0.4. Median with interquartile range (IQR) was used to describe continuous variables. Frequency analysis with percentage was employed to describe categorical variables. In data management, monthly individual income was categorised into quintiles ranging from Q1 (poorest) to Q5 (richest). In regards to the Poverty and Inequality Report 2019 [25], we applied the cut-off point from the decile income in 2013. A Mann-Whitney U test and Chi-square were used to compare the demographic characteristics of the subjects with alcohol-related harms to children. Finally, a multivariable binary logistic regression model was applied to assess the determinants in regards to alcohol related harm to children. Potential confounders (i.e., demographic characteristics, and presence of binge or frequent drinker in household) were included in the initial model. Backward stepwise refinement was performed. A p-value less than 0.05) indicated statistical significance.

### Ethical consideration

ThaiHealth and International Health Policy Program (IHPP), Ministry of Public Health allowed the investigators to analyse the original dataset to answer this research objectives. We have applied protocols for analyses of anonymised secondary data. Ethical clearance for secondary data analysis was given by the Human Research Ethics Committee of the Faculty of Medicine, Prince of Songkla University, Thailand (Ref No. 64-139-9-1).

## Results

### The number of identified alcohol-related harms to children

Among 925 participants who completed the survey **(Table 1)**, there were 147 (15.89%) participants who reported that their children had been affected by someone’s drinking as per 1 or 2 problem domains in the last 12 months. The top three problems were serious domestic violence (7.45%), verbal abuse (7.25%), and financial support for children (5.27%). Less than 1% of the participants reported that their children were a protection agency or that family services were involved because of someone’s drinking.

**Table 1:** The number of identified alcohol-related harms to children.

Domain	Number of respondents	Number of events	Percent prevalence of event (95%CI)
**Because someone’s drinking (in the last 12 months)**			
**Any problems (at least one problem below)**	925	147	15.89 (13.59, 18.41)
Did children witness serious violence in the home?	926	69	7.45 (5.84, 9.34)
Were children yelled at, criticized, verbally abused?	952	69	7.25 (5.68, 9.08)
Were there not enough money for the things needed by children?	930	49	5.27 (3.92, 6.91)
Were children abandoned in an unsupervised or unsafe situation?	929	33	3.55 (2.46, 4.95)
Were physically hurt?	930	16	1.72 (0.99, 2.78)
Was child protection agency or family services called?	931	1	0.11 (0.003, 0.60)

CI: Confidence interval.

### The relationship between number of identified alcohol-related harm and relationship

**Table 2** shows the answer that respondents gave about who was the perpetrator regarding each type of violence associated with alcohol consumption. The perpetrators cannot be identified in regard to about half of the top three specific alcohol-relate harms (i.e., domestic violence, verbal abuses, and financial support problems). However, if the unspecified part is eliminated, it is found that most of the cases are caused by their parents (34.8% in domestic violence, 29.0% in verbal abuses, and 28.5% in financial support problems), except for physical hurt which was caused by their father’s/mother’s side cousin.

**Table 2:** The relationship between number of identified alcohol-related harm and relationship.

Domain	Relationship, n (row %)						
	Parent Father/Mother	Father’s/Mother’s side cousin	Friend/Teacher	Sibling	Others	Not specified	Total
**Because someone’s drinking (in the last 12 months)**							
**Any problems (at least one problem below)**	33 (22.5)	23 (15.6)	4 (2.7)	1 (0.7)	21 (14.3)	65 (44.2)	147
Did children witness serious violence in the home?	24 (34.8)	12 (17.4)	0	0	5 (7.2)	28 (40.6)	69
Were children yelled at, criticised, verbally abused?	20 (29.0)	15 (21.7)	3 (4.3)	0	7 (10.1)	24 (34.8)	69
Were there not enough money for the things needed by children?	14 (28.5)	8 (16.3)	1 (2.1)	1 (2.1)	5 (10.2)	20 (40.8)	49
Were children abandoned in an unsupervised or unsafe situation?	8 (24.2)	8 (24.2)	3 (9.1)	0	7 (21.2)	7 (21.2)	33
Were physically hurt?	4 (25.0)	5 (31.3)	1 (6.3)	0	4 (25.0)	2 (12.5)	16
Was child protection agency or family services called?	0	0	0	0	0	1 (100)	1

### Relationship between alcohol-related harms to children, demographic characteristics and presence of drinker in household

There were no significant differences in the participants between the two groups, except for age and the presence of a binge or frequent drinker in the household **(Table 3)**. The children around the younger participants had a higher probability of being exposed to alcohol related violence than those around older participants. Furthermore, there was a higher prevalence of binge drinkers (93.9% vs. 70.2%), monthly or less drinkers (28.6% vs. 26.3%), and weekly drinkers (25.2% vs. 16.5%) in those who had alcohol-related harms in their households vs. those who didn’t.

**Table 3:** Relationship between alcohol-related harms to children, demographic characteristics and presence of drinker in household.

Factors	Any alcohol-related harms, n (%)		Total	P value
	No (n = 778)	Yes (n = 147)		
**Gender**				
Male	307 (39.5)	53 (36.1)	360 (38.9)	0.494_a_
Female	471 (60.5)	94 (63.9)	565 (61.1)	
**Age (years)**				
Median (IQR)	44 (35,53)	42 (33,50)	47 (37,56)	0.004_b_*
18–29	96 (12.3)	27 (18.4)	123 (13.3)	0.023_a_*
30–59	586 (75.3)	114 (77.6)	700 (75.7)	
≥60	96 (12.3)	6 (4.1)	102 (11)	
**Number of household member**	4 (4,6)	4 (4,6)	4 (3, 5)	0.601_b_
**Education of respondent**				
Pre-Secondary	367 (47.3)	76 (51.7)	443 (48)	0.366_a_
Secondary	292 (37.6)	55 (37.4)	347 (37.6)	
Post-Secondary	117 (15.1)	16 (10.9)	133 (14.4)	
**Residential area**				
Rural	300 (38.6)	65 (44.2)	365 (39.5)	0.232_a_
Urban	478 (61.4)	82 (55.8)	560 (60.5)	
**Monthly income quintile**				
Q1 (<3,231.5 THB)	230 (35.7)	70 (51.9)	300 (38.5)	0.007_a__*_
Q2 (3,231.5–5,002.4 THB)	147 (22.8)	28 (20.7)	175 (22.5)	
Q3 (5,002.5–7,494.9 THB)	83 (12.9)	10 (7.4)	93 (11.9)	
Q4 (7,495.0–12,419 THB)	110 (17.1)	18 (13.3)	128 (16.4)	
Q5 (≥ 12,420 THB)	74 (11.5)	9 (6.7)	83 (10.7)	
**Presence of a binge drinker in household**				
No	232 (29.8)	9 (6.1)	241 (26.1)	< 0.001_a_*
Yes	546 (70.2)	138 (93.9)	684 (73.9)	
**Presence of a frequent drinker in household**				
Not in previous year	445 (57.3)	68 (46.3)	513 (55.5)	0.017_a_*
Monthly or less	204 (26.3)	42 (28.6)	246 (26.6)	
Weekly	128 (16.5)	37 (25.2)	165 (17.9)	

IQR: Interquartile range, THB = Thai Baht (1 USD = 30 THB).

_a_ Chi-square test

_b_ Mann-Whitney U test.

### Factors influencing alcohol-related harms to children

After adjusting for potential confounders including gender, age, education of response, residential area, monthly individual income **(Table 4)**, people aged more than 60 years old are less likely to report alcohol-related harm to children than people aged between 18 to 29 years (adjusted odds ratio [AOR] 0.19, 95% confident interval [Cl]: 0.06–0.58).

**Table 4:** Factors influencing alcohol-related harms to children.

Factor	Crude OR (95%CI)	Adjusted OR (95%CI)
**Gender**		
Male	1	1
Female	1.21 (0.82, 1.79)	1.62 (0.99, 2.61)
**Age (years)**		
18–29	1	1
30–59	0.68 (0.42, 1.12)	0.64 (0.36, 1.12)
≥60	0.23 (0.08, 0.62)	0.19* (0.06, 0.58)
**Number of household member**	1.10 (0.99, 1.22)	1.08 (0.97, 1.20)
**Education of respondent**		
Pre-Secondary	1	1
Secondary	0.84 (0.56, 1.25)	0.74 (0.46, 1.19)
Post-Secondary	0.69 (0.38, 1.25)	0.83 (0.40, 1.70)
**Residential area**		
Rural	1	1
Urban	0.89 (0.61,1.29)	0.88 (0.59, 1.32)
**Monthly income quintile**		
Q1 (<3,231.5 THB)	1	1
Q2 (3,231.5–5,002.4 THB)	0.63 (0.39, 1.02)	0.62 (0.37, 1.03)
Q3 (5,002.5–7,494.9 THB)	0.40* (0.19, 0.80)	0.37* (0.17, 0.77)
Q4 (7,495.0–12,419 THB)	0.54* (0.31, 0.95)	0.60 (0.32, 1.13)
Q5 (≥ 12,420 THB)	0.41* (0.19, 0.85)	0.47 (0.20, 1.08)
**Presence of a binge drinker in household**		
No	1	1
Yes	5.36* (2.67, 10.77)	4.75* (2.33, 9.66)
**Presence of a frequent drinker in household**		
Not in previous year	1	1
Monthly or less	1.15 (0.75, 1.78)	1.05 (0.66, 1.67)
Weekly	1.61 (0.99, 2.60)	1.92* (1.06, 3.47)

OR: Odds ratio, THB = Thai Baht (1 USD = 30 THB).

* P-value < 0.05.

Households with a binge drinker tended to have higher risk for alcohol-related harm to children compared with households without a binge drinker (AOR 4.75, 95%Cl: 2.33–9.66). In addition, the presence of a weekly drinker in the household results to a higher risk of alcohol-related harm to children, compared with a household that has an ex-drinker that is abstinent for 12-months (AOR 1.92, 95%Cl: 1.06–3.47). The interaction between the presence of binge and frequent drinkers was not significant (p>0.05).

## Discussion

### Principal finding and previous studies

We discovered that about 16 percent of Thai children have experienced at least one or more problems because of someone else’s drinking. Most self-reported problems were serious domestic violence and verbal abuse. Most of the problems affected by children were caused mostly by their parents. However, in almost half of the recorded problems affecting children, the offenders are not identified. Interestingly, children whose family members consumed alcohol (i.e., binge or weekly drinking) were more likely to have problems than those whose family members did not drink alcohol.

Domestic violence was a global major public health concern [26,27]. In 2006, the UNICEF (United Nations Children’s Fund) estimated that between 133 million and 275 million children around the world witness frequent domestic violence each year [28]. In Australia, alcohol was found in 47.6 percent or 20,489 cases of substantiated cases of domestic violence since 2001–2005 [29] and there were three percent of 1,142 respondents in the parental subset who had parental responsibility for 2,457 children and reported that children were exposed to domestic violence [11]. Children who witness domestic violence are more likely to develop post-traumatic stress disorder, violent behavior, anxiety, impaired development, difficulty interacting with peers, academic problems, and have a higher incidence of substance misuse [30]. As a result, domestic violence in children is a serious issue that should not be ignored.

Another common finding in this study was the presence of verbal abuse, which had a similar percentage to domestic violence. Verbal abuse is defined as the intentional causing of emotional pain through yelling, screaming, threatening, humiliating, infantilising, or causing fear [17,31]. It is some of the most common and often overlooked forms of child abuse [32]. In 2012, nine percent of 2,457 children in Australia were exposed to verbal abuse because of other people drinking alcohol [11].

According to data from Australia’s health 2012 report, the majority of abusers (80.9%) were the children’s parents [33], we found similar results in our study. Domestic violence, verbal abuse, and child support issues were associated with their parents [33]. Parents’ history of physical abuse, being a young parent, witnessing domestic violence, and having poor self-control were the risk factors for child abuse [34]. Furthermore, there was an association between the type of family and child maltreatment. For instance, children from single-parent households or families were more likely to be abused than those from married households. Parental background is also key and in regards to it there are crucial risk factors associated to child related harm such as young age, socioeconomic factors, adverse childhood experiences, low educational achievement, and having a previous psychiatric history [35]. While there were strong relationships between alcohol and child maltreatment. Hazardous alcohol use by parents can impair their sense of personal responsibility and reduce a lot of time and resources available for the child. Children’s basic needs may be neglected in such cases [34]. In this study, we also discovered that the presence of a regular drinker in the household tends to increase the risk of harm to children. Similarly, Yang and Kramer has reported that exposure to weekly paternal alcohol consumption was related with negative behavioural development in their children [36].

When other influencing factors for alcohol-related harms to children were considered, we discovered that the children around the younger participants had a higher probability of being exposed to alcohol related violence than those around older participants. Many studies have found that there is a higher rate of abuse suffered by the children of young mothers vs. the children of older mothers [37]. However, Schumacher et al. discovered no consistency in regards to parental age being a risk factor for certain types of maltreatment [38]. Harmful parental alcohol use has been linked to other parenting issues such as poor mental health and anti-social personality traits, both of which are known to increase the likelihood of poverty and child maltreatment [39].

### Study limitations

There were some limitations in this study. Firstly, the questionnaire from the WHO-Thai Health project did not ask for the age of the children. Thus, we were unable to determine the age of individual children affected by alcohol. We only know that all children in the report were under the age of 18. Secondly, this study cannot estimate the time duration or frequency that children were abused because of someone drinking alcohol. Finally, the study cannot predict the long-term effects that can occur in children who are victims of abuse related to perpetrators consuming alcohol.

## Conclusion

This study found that children whose family members had consumed alcohol (i.e., weekly drinker or binge drinker) were significantly adversely affected. The common problems were domestic violence and verbal abuse. Most of the problems that affected the children were mostly caused by the parents. However, in regards to almost half of the problems affecting children, the offenders are not identified.

## References

[1] World Health Organization. Global status report on alcohol and health 2018. Geneva: World Health Organization; 2018.

[2] Richardson A, Budd T. Young adults, alcohol, crime and disorder. Criminal Behaviour and Mental Health. 2003;13: 5–16. doi: 10.1002/cbm.527 14624268

[3] Waleewong O, Laslett A-M, Chenhall R, Room R. Harm from others’ drinking-related aggression, violence and misconduct in five Asian countries and the implications. International Journal of Drug Policy. 2018;56: 101–107. doi: 10.1016/j.drugpo.2018.03.015 29621741

[4] Thavorncharoensap M, Teerawattananon Y, Yothasamut J, Lertpitakpong C, Chaikledkaew U. The economic impact of alcohol consumption: a systematic review. Subst Abuse Treat Prev Policy. 2009;4: 20–20. doi: 10.1186/1747-597X-4-20 19939238 PMC2791094

[5] Griswold MG, Fullman N, Hawley C, Arian N, Zimsen SR, Tymeson HD, et al. Alcohol use and burden for 195 countries and territories, 1990–2016: a systematic analysis for the Global Burden of Disease Study 2016. The Lancet. 2018;392: 1015–1035. doi: 10.1016/S0140-6736(18)31310-2 30146330 PMC6148333

[6] Moan IS, Storvoll EE, Sundin E, Lund IO, Bloomfield K, Hope A, et al. Experienced Harm from Other People’s Drinking: A Comparison of Northern European Countries. Subst Abuse. 2015;9: 45–57. doi: 10.4137/SART.S23504 26609236 PMC4648612

[7] Laslett A-M, Callinan S, Pennay A. The increasing significance of alcohol’s harm to others research. Drugs and Alcohol Today. 2013;13: 163–172. doi: 10.1108/dat-11-2012-0010

[8] Room R, Ferris J, Laslett A-M, Livingston M, Mugavin J, Wilkinson C. The drinker’s effect on the social environment: a conceptual framework for studying alcohol’s harm to others. Int J Environ Res Public Health. 2010/04/21 ed. 2010;7: 1855–1871. doi: 10.3390/ijerph7041855 20617064 PMC2872341

[9] World Health Organization. Global status report on alcohol and health 2014. World Health Organization; 2014.

[10] Kaplan LM, Nayak MB, Greenfield TK, Karriker-Jaffe KJ. Alcohol’s Harm to Children: Findings from the 2015 United States National Alcohol’s Harm to Others Survey. J Pediatr. 2017/02/15 ed. 2017;184: 186–192. doi: 10.1016/j.jpeds.2017.01.025 28215936 PMC5403548

[11] Laslett A-M, Ferris J, Dietze P, Room R. Social demography of alcohol-related harm to children in Australia. Addiction. 2012;107: 1082–1089. doi: 10.1111/j.1360-0443.2012.03789.x 22229839

[12] Connor J, Kydd R, Rehm J, Shield K. Alcohol-attributable burden of disease and injury in New Zealand: 2004 and 2007. Research report commissioned by the Health Promotion Agency; 2013.

[13] Laslett A-ML, Dietze PM, Room RGW. Carer Drinking and More Serious Child Protection Case Outcomes. British Journal of Social Work. 2012;43: 1384–1402. doi: 10.1093/bjsw/bcs052

[14] Yu J. The association between parental alcohol-related behaviors and children’s drinking. Drug and Alcohol Dependence. 2003;69: 253–262. doi: 10.1016/s0376-8716(02)00324-1 12633911

[15] Cussen T, Bryant W. Domestic family homicide in Australia. 2015.

[16] O’Donnell M, Maclean MJ, Sims S, Morgan VA, Leonard H, Stanley FJ. Maternal mental health and risk of child protection involvement: mental health diagnoses associated with increased risk. Journal of Epidemiology and Community Health. 2015;69: 1175–1183. doi: 10.1136/jech-2014-205240 26372788

[17] Substance Abuse and Mental Health Services Administration. Children of Alcoholics: A Guide to Community Action. 2004. doi: 10.1016/j.biopsych.2004.03.005 15556113

[18] Manning V, Best DW, Faulkner N, Titherington E. New estimates of the number of children living with substance misusing parents: results from UK national household surveys. BMC Public Health. 2009;9: 377–377. doi: 10.1186/1471-2458-9-377 19814787 PMC2762991

[19] Callinan S, Livingston M. Increases in alcohol consumption in middle-income countries will lead to increased harms. The Lancet. 2019;393: 2471–2472. doi: 10.1016/S0140-6736(18)33002-2 31076175

[20] Jokinen T, Alexander EC, Manikam L, Huq T, Patil P, Benjumea D, et al. A Systematic Review of Household and Family Alcohol Use and Adolescent Behavioural Outcomes in Low- and Middle-Income Countries. Child Psychiatry Hum Dev. 2021;52: 554–570. doi: 10.1007/s10578-020-01038-w 32785812 PMC8238760

[21] Laslett A-M, Mojica-Perez Y, Waleewong O, Hanh HTM, Jiang H. Harms to children from the financial effects of others’ drinking. International Journal of Drug Policy. 2021;94: 103254. doi: 10.1016/j.drugpo.2021.103254 33887675

[22] Laslett A-M, Rankin G, Waleewong O, Callinan S, Hoang HTM, Florenzano R, et al. A Multi-Country Study of Harms to Children Because of Others’ Drinking. J Stud Alcohol Drugs. 2017;78: 195–202. doi: 10.15288/jsad.2017.78.195 28317499 PMC5554100

[23] Waleewong O, Jankhotkaew J, Thamarangsi T, Chaiyasong S. Prevalence of harm from others’ alcohol drinking and the relationships with demographics and the respondents’ drinking behaviors in Thailand. Journal of Substance Use. 2017;22: 605–611. doi: 10.1080/14659891.2017.1283451

[24] Callinan S, Laslett A-M, Rekve D, Room R, Waleewong O, Benegal V, et al. Alcohol’s harm to others: an international collaborative project. 2016.

[25] Office of the National Economics and Social Development Council, Thailand. Poverty and Inequality Report 2019 in Thailand. Available: https://www.nesdc.go.th/ewt_dl_link.php?nid=10857&filename=social.

[26] Watts C, Zimmerman C. Violence against women: global scope and magnitude. The lancet. 2002;359: 1232–1237. doi: 10.1016/S0140-6736(02)08221-1 11955557

[27] Devries KM, Mak JY, Garcia-Moreno C, Petzold M, Child JC, Falder G, et al. The global prevalence of intimate partner violence against women. Science. 2013;340: 1527–1528. doi: 10.1126/science.1240937 23788730

[28] Pinheiro PS. World report on violence against children. Geneva: United Nations Publ; 2006.

[29] Laslett A-M, Alcohol Education and Rehabiliation Foundation, Turning Point Alcohol and Drug Centre (Vic.). The range and magnitude of alcohol’s harm to others: beyond the drinker: alcohol’s hidden costs. Fitzroy, Vic.: AER Foundation; 2010.

[30] Government Offices of Sweden. Domestic violence—a public health issue. [cited 23 Aug 2021]. Available: https://www.government.se/reports/2014/06/domestic-violence—a-public-health-issue/.

[31] Tatara T, Kuzmeskus LB, Duckhorn E, Bivens L, Thomas C, Gertig J, et al. The National Elder Abuse Incidence Study: 136.

[32] Rerkswattavorn C, Chanprasertpinyo W. Prevention of child physical and verbal abuse from traditional child discipline methods in rural Thailand. Heliyon. 2019;5: e02920. doi: 10.1016/j.heliyon.2019.e02920 31867455 PMC6906657

[33] Australian Institute of Health and Welfare. Australia’s health 2012 [electronic resource]: the thirteenth biennial health report of the Australian Institute of Health and Welfare. Canberra: The Institute; 2012. Available: http://www.aihw.gov.au/WorkArea/DownloadAsset.aspx?id=10737422169.

[34] World Health Organization. Child maltreatment and alcohol. 2020. Available: https://www.who.int/violence_injury_prevention/violence/world_report/factsheets/fs_child.pdf. doi: 10.1016/j.alcohol.2020.05.004 32561311 PMC7958146

[35] Okonya R. The Role of Family Structure in the Abuse of Children. Public Health. 2018; 127.

[36] Yang S, Kramer MS. Paternal alcohol consumption, family transition and child development in a former Soviet country. International Journal of Epidemiology. 2012;41: 1086–1096. doi: 10.1093/ije/dys071 22586132

[37] Black DA, Smith Slep AM, Heyman RE. Risk factors for child psychological abuse. Aggression and Violent Behavior. 2001;6: 189–201. doi: 10.1016/S1359-1789(00)00022-7

[38] Schumacher JA, Slep AMS, Heyman RE. Risk factors for child neglect. Aggression and Violent Behavior. 2001;6: 231–254. doi: 10.1016/S1359-1789(00)00024-0

[39] Cancian M, Yang M-Y, Slack KS. The effect of additional child support income on the risk of child maltreatment. Social Service Review. 2013;87: 417–437.

